# A Comprehensive Review of Arsenic Exposure and Risk from Rice and a Risk Assessment among a Cohort of Adolescents in Kunming, China

**DOI:** 10.3390/ijerph15102191

**Published:** 2018-10-08

**Authors:** Noelle Liao, Edmund Seto, Brenda Eskenazi, May Wang, Yan Li, Jenna Hua

**Affiliations:** 1 Department of Preventive Medicine, Keck School of Medicine, University of Southern California, Los Angeles, CA 90033, USA; noelleliao@alumni.usc.edu; 2 Department of Environmental & Occupational Health Sciences, School of Public Health, University of Washington, Seattle, WA 98195, USA; eseto@uw.edu; 3 Department of Maternal and Child Health, School of Public Health, University of California, Berkeley, CA 94720, USA; eskenazi@berkeley.edu; 4 Department of Community Health Sciences, School of Public Health, University of California, Los Angeles, CA 90095, USA; maywang@ucla.edu; 5 Kunming Medical University, Kunming 650221, Yunnan, China; yanli20021965@21cn.com; 6 Stanford Prevention Research Center, School of Medicine, Stanford University, Stanford, CA 94305, USA

**Keywords:** arsenic, cancer, rice, concentration, diet, risk assessment, exposure, China, Asia, adolescents

## Abstract

Inorganic arsenic (iAs) is carcinogenic and highly concentrated in rice. Dietary exposure to iAs is concerning among adolescents due to their developmental stage and iAs’s long-latency effects. This paper aimed to assess iAs exposure from rice and related lifetime cancer risks (LCR) among adolescents in Kunming, China. A comprehensive literature review of iAs levels in rice and LCR in humans was also conducted. Average daily consumption of rice (ADC) was estimated from 267 adolescents (15–18 years). Rice samples obtained from 6 markets were analyzed for iAs concentration (AC). Estimated daily intake (EDI) of iAs was calculated using ADC, AC, and average body weight (BW). Lifetime Cancer Risk (LCR) was calculated using EDI and U.S. EPA derived iAs oral slope factor. The AC was 0.058 mg/kg and the average BW and ADC were 67.5 kg and 410 g/day for males and 55.5 kg and 337 g/day for females. The EDI and LCR were 3.52 × 10^−4^ mg/kg-BW/day and 5.28 × 10^−4^ for both males and females, with LCR 5 times above the U.S. LCR upper limit of 1.0 × 10^−4^. While the AC was below the Chinese maximum contaminant level of 0.2 mg/kg, study results indicated that Kunming adolescents may be at increased risk for iAs-related cancers.

## 1. Introduction

Exposure to toxic contaminants in food is presumed to be one of the major public health challenges for the 21st century [1]. Arsenic (As) is especially prevalent in the environment and can easily enter the food system through contaminated soil or water. Of arsenic’s two chemical forms (organic and inorganic), inorganic arsenic (iAs) is classified by the International Agency for Research on Cancer (IARC) as a non-threshold (even small doses may provide some cancer risk) class 1 human carcinogen and is associated with skin, lung, liver, kidney, and bladder cancers [2]. Among As species, i-AsIII (arsenite) is the most abundant species found in rice and also the most toxic to humans [3,4].

Arsenic has no single major mode of action in the human body [5]. A few of the well-documented mechanisms describing arsenic’s influence on cancer are (1) increasing the generation of hydrogen peroxide and superoxide anions; (2) interacting with cysteine residues in zinc finger domains, ultimately leading to loss of protein function; (3) deregulating cell proliferation and inducing epigenetic alterations [6]. Because cancer risk may be non-linear, a safe level cannot be determined by extrapolating risk from high dose exposures [6].

Flooded rice fields and the anaerobic nature of paddy soils facilitate the buildup of arsenic in the rice crop compared to other agricultural crops [2]. Rice can accrue up to 10–20 times more arsenic than wheat or barley because silica and phosphate transporters in the rice crop effectively move iAs up into the rice grain [7]. According to the Food and Agriculture Organization of the United States (FAO), China is the highest rice producing and consuming country in the world and accounts for a third of the global supply of rice [8,9]. Rice grown in Asia has also been found to have higher iAs content than rice grown in the U.S. [10]. An estimated 57–96% of the total arsenic measured in Chinese-produced rice has been found to be of the inorganic form, which stresses the importance of conducting iAs risk assessments in China [11]. Nonetheless, estimating iAs exposure from rice is difficult in China due to the vast size of the country, its variable geology, and the country’s diverse dietary patterns among sub-populations [11]. These factors strongly emphasize the importance of doing regional risk assessment studies in China.

Until recently, official food monitoring across the world considered only total As in rice (not inorganic As species) and lately iAs exposure and its health effects have become national and international concerns [12]. Likewise, many studies rarely measure iAs in rice directly and instead only estimate iAs using a fraction of the total arsenic measured [13]. Related studies also lack the use of individual-level data and/or do not estimate cancer risk using rice samples, individual consumption rates, and body weights from the same, specific region. These factors are all critical for an accurate health risk assessment of iAs. Furthermore, certain populations may be particularly at risk. For example, because of the long latency period (approximately 25 years) of iAs-related cancer, children have a greater potential for long-term exposure [14]. Children, including adolescents, are also in a critical window of development, may have a high calorie per unit body weight diet compared to adults, and tend to be more exposed to contaminants unique to specific foods due to selective and less diverse dietary patterns [15].

Therefore, the aims of this study are to: (1) conduct a comprehensive literature review of recent studies published after 2010 that measured arsenic concentrations in rice and/or arsenic exposure levels in humans, or conducted arsenic-related cancer risk assessments; (2) estimate lifetime cancer risk among a cohort of adolescents living in Kunming, China using directly measured iAs levels in locally sampled rice and individual-level rice consumption data of Kunming adolescents; and (3) compare this study cohort’s iAs exposure rates and lifetime cancer risks to that of other populations in the published literature.

## 2. Materials and Methods

### 2.1. Literature Review

A comprehensive literature review was conducted using Google Scholar and PubMed with key search terms including arsenic, rice, concentration, cancer, exposure, diet, risk assessment, China, Asia, and adolescents. Papers excluded in this manuscript’s tables were reviews, papers published before 2010 in order to provide a more concise review of more current As levels in rice and exposure levels in populations, and arsenic risk assessments with outcomes other than cancer. However, papers with an outcome other than cancer that had measurements of arsenic concentrations in rice, as well as having China or Asia as the study locations, were included in the assessment in order to account for those Asian risk assessment studies that measured As levels in rice.

### 2.2. Rice Sample Collection and Lab Analysis

Short-grain white rice samples were collected from six Kunming markets, varying by location and market type, to represent foods purchased in different Kunming neighborhoods and by different financial preferences, such as from a high-end market selling organic food and at a Carrefour (hypermarket). The collected rice samples were sourced from growing regions in Northern China, as is most of the rice sold in Kunming.

Samples were sent to the commercial laboratory Merieux Nutrisciences located in Ningbo, China for analysis. The laboratory received seven labeled samples from seven different locations, two of which were from the same location in order to assess the reliability of the lab’s measurement procedures. The lab used the 2009 updated version of the GB/T 5009.11-2003 method for determination of abio-arsenic (iAs) in the received rice samples. By means of hydride generation atomic fluorescence spectrophotometry, iAs was extracted in the form of chloride and separated from organic arsenic during water bathing with HCL, and its concentration was measured [16]. Specifically, 2.50 g of solid sample was blended with 20 mL of HCL (1 + 1) solution and put into a 60 °C water bath for 18 h and then filtered. 4 mL of filtrate was mixed with 1 mL of KI-thiourea mixed solution and 8 drops of *n*-octanol, diluted to volume with water, and settled for 10 min to determine iAs concentration. Standard series of iAs determination used 1 μg/mL As3+ standard solution [16].

### 2.3. Study Population

Adolescents (267, 45.7% males and 54.3% females) aged 15–18 years old and all of Han ethnicity were recruited from two local Kunming high schools in 2015 using convenience sampling methods. The schools had a wide catchment of students who lived in various regions of the city. Study participants completed 72-h dietary recalls which captured their diets over two weekdays and a weekend day (Thursday, Friday and Saturday). Participants were trained on estimating portion sizes and how to fill out a 72-h dietary recall. Participants’ weights were also measured in light clothing without shoes to the nearest 0.1 km using an electronic scale. All human subject data collection procedures were approved by the Institutional Review Boards of UC Berkeley and Kunming Medical University (2014-03-6097).

### 2.4. Exposure Estimation and Cancer Risk Calculation

Average daily consumption rates (ADC) of white rice (g/day) were estimated using the 72-h recalls for both males and females separately. Estimated daily intake (EDI) of iAs from rice consumption (mg/kg-BW/day) was calculated using the ADC, average concentration (AC) of iAs in sampled rice, and average body weight of the adolescents (BW) [17]:

Estimated daily intake:

EDI = (AC × ADC)/BW,(1)

Lifetime Cancer Risk (LCR), the probability of excess lifetime cancer risk, was calculated using the EDI and the U.S. EPA derived iAs oral slope factor (SF), 1.5 (mg/kg)/day [5]. The oral slope factor is the plausible upper-bound estimate of the probability of a response per unit intake of a chemical over a lifetime and refers to a linear, non-threshold model of risk [7]. Lifetime cancer risk assumes daily exposure (365 days of the year) over one’s entire lifetime.

Lifetime cancer risk:

LCR = EDI × SF,(2)

Stata version 15.0 (StataCorp, College Station, TX, USA, 2017) was used to calculate the average daily consumption rates of rice and mean body weights of males and females in the study cohort. A *t*-test was conducted to compare the ADCs and BWs between males and females. A *p*-value of less than or equal to 0.05 was selected to indicate statistical significance.

## 3. Results

### 3.1. Literature Review

Fifty four papers were screened and 38 fitted the table inclusion criteria. Only seven studies focused on Southern China and the other eight Chinese studies focused on other regions or multiple provinces throughout China. It was estimated that Southern China has 2.5 times higher rice consumption rates than Northern China and higher concentrations of arsenic in rice [11,18]. Of those seven studies focusing on Southern China, five studies had separate risk assessment analyses for children and one study for adolescents. Thirteen out of the 38 studies included in the review tables (Table 1 and Table 2) directly measured iAs in rice. Huang et al. conducted the only regional risk assessments in China using individual-level data [19]. The other studies conducted in China pulled consumption data from previously published literature or used nation-wide dietary or ecological-level data. Given the varied dietary patterns among different regions of China and among different age groups, using averaged nation-wide data may not be reflective of unique regional differences.

**Figure 1:**
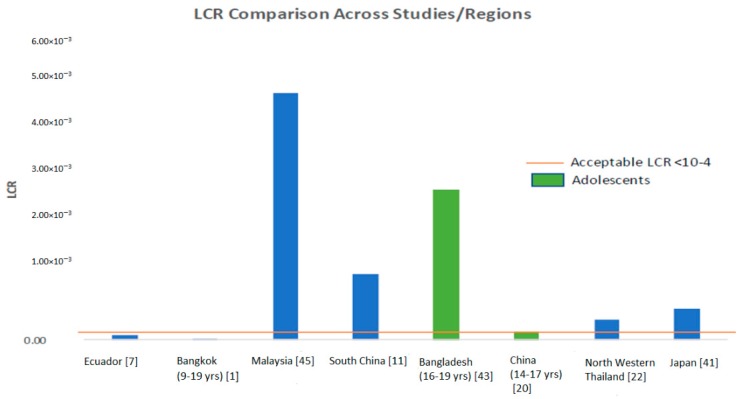
Comparison of LCR values from different studies and regions.

Table 1 summarizes the studies that assessed arsenic concentrations (total As and/or iAs) in rice and did not include a cancer risk assessment. Chen et al. tested 160 rice samples from local markets in 20 provinces in China and the results indicated average iAs levels (mg/kg) of 0.054 across all regions, 0.058 in Southern China, 0.61 in Middle China, 0.042 in Eastern China, and 0.048 in Northern China [20]. Table 2 summarizes the studies that conducted cancer risk assessments of arsenic. Some of the arsenic risk assessment studies do not calculate LCR and instead compare their population’s EDI to the previous provisional tolerable daily intake (PTDI) of 2.1 × 10^−3^ mg/kg-BW/day recommended by the WHO [21]. Notably, the PTDI has been withdrawn because it is no longer considered health protective due to the BMDL_0.5_ of 3.0 μg/kg-BW/day (lower 95% confidence limit on the benchmark dose for a 0.5% increased incidence of lung cancer) residing in its range (2 × 10^−3^–7 × 10^−3^ mg/kg-BW/day) [10]. The benchmark dose is the dose that is associated with a specific change in an adverse response compared to the response in unexposed persons [22]. We note that if data on adolescents were specifically provided in a study, we presented those in the table. As indicated in Table 2, iAs exposures and related LCRs were particularly high for Asian countries such as China, India, Pakistan, Bangladesh and Malaysia and lower for Europe and the Americas, with adolescents in Bangladesh having exceptionally high LCRs (Figure 1). Figure 1 compares LCR values in the risk assessment studies (Table 2) that reported LCRs. It serves as a graphical demonstration of the diversity of cancer risks and the populations that are potentially most at risk. The U.S. EPA LCR acceptable upper limit is 1.0 × 10^−4^ and it is apparent that many Asian countries and even adolescents in those populations greatly exceeded the upper limit [5].

**Figure 2:**
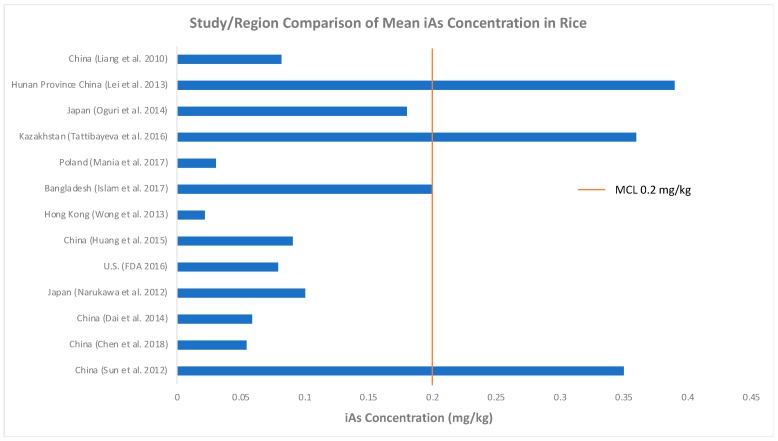
Comparison of iAs concentrations in rice in different studies and regions.

Figure 2 compares studies that directly measured iAs concentrations in rice. The Chinese maximum contaminant level (MCL) for iAs in polished rice is 0.2 mg/kg [48] and the graph displays that many studies measured iAs concentrations well below the MCL, which may or may not be health protective depending on corresponding rice consumption rates.

### 3.2. iAs Concentrations in Sampled Rice

The iAs concentrations measured in the Kunming samples ranged from 0.045–0.076 mg/kg and are below the Chinese MCL. Of the two samples from the same location (one sample blinded to the commercial laboratory), total arsenic concentrations were 0.080 mg/kg for both samples and iAs concentrations were 0.054 mg/kg and 0.050 mg/kg. This provided assurance of the reliability of the lab measurement procedures. Interestingly, the sample from the high-end market selling organic food had the second highest level of iAs (0.067), which demonstrates that price and quality of products may not be a factor that reduces arsenic contamination in food products. These seven measurements averaged to be 0.058 mg/kg (markedly the same mean value for Southern China measured by Chen et al. [20]). We applied this value for our current risk assessment for both males and females.

### 3.3. Dietary Rice Consumption

Among the study participants, males consumed on average 409.9 g/day of rice, 22% higher than that of females (336.6 g/day). *t*-test results (*p* < 0.001) indicated that males and females differed significantly in their average daily consumption rate of rice. Due to uncertainties in assessing rice content in popular rice products such as rice cakes and rice snacks, calculated consumption rates did not take into account these products. Thus, we may have underestimated the g/day of rice consumption in the study participants.

### 3.4. Estimated Daily Intake of iAs and Cancer Risk

Table 3 displays the values used to calculate the EDI and LCR for adolescents in this study. The mean BW for males was 67.5 kg (range 44.0–115.9 kg; SD 14.4 kg) and for females it was 55.5 kg (range 37.5–99.4 kg; SD 10.6 kg). *t*-test results (*p* < 0.001) indicating that males and females differed significantly in their body weights. The EDI for males was estimated to be 3.52 × 10^−4^ mg/kg-BW/day (max. 5.00 × 10^−4^ mg/kg-BW/day) and for females was estimated to be 3.52 × 10^−4^ mg/kg-BW/day (max. 5.35 × 10^−4^ mg/kg-BW/day).

The calculated average LCR for both males and females using the cancer slope factor of 1.5 (mg/kg)/day was 5.28 × 10^−4^ (max. 7.51 × 10^−4^), which is 5 times above the upper limit of the U.S. EPA LCR of 1.0 × 10^−4^. Due to differences in body weight and ADC, the calculated maximum LCR for females was 8.02 × 10^−4^, which is eight times above the upper limit of the LCR.

## 4. Discussion

### 4.1. Risk among Kunming Adolescents

The mean iAs concentration in this study’s rice samples was 0.058 mg/kg and the lifetime cancer risk for both males and females in this study cohort was estimated to be 5.28 × 10^−4^. Even though measured iAs concentrations in the sampled rice in this study were below the Chinese MCL, the high consumption rates of rice in this cohort are driving LCR values beyond the LCR acceptable upper limit. Our cohort’s LCR was over three times greater than the LCR of adolescents in Jiang et al., which used consumption data from the Survey of the Nutrition and Health Status (NHS) of the Chinese People in 2002 to estimate iAs exposure from all foods measured, not just rice. In that study, the authors did not directly measure iAs, but used conversion factor of 20.3% to estimate iAs from total arsenic [38]. Awata et al. used the NHANES (2001–2012) data which was the first NHANES cycle to oversample Asians in the U.S. and found that Chinese adolescents (19–21 years old) had iAs dietary intakes of 8 × 10^−5^ mg/kg-BW/day, which is 4.4 times lower than that of our study cohort [49]. This may be due to different eating habits between Chinese and Chinese-American adolescents.

### 4.2. Arsenic in Rice and Cancer Risk

The current Chinese MCL of 0.2 mg/kg, reflective of the maximum level set by the Codex Alimentarius Commission, was raised from the 2005 Chinese MCL of 0.15 mg/kg [48]. Given the high consumption rates of rice in China, increasing the MCL may inadvertently increase the risk of iAs-related cancer in this country. Based on the adolescent’s rice consumption data collected for this study, if the cohort is being exposed at MCL of 0.2 mg/kg, the EDI and LCR estimations would be 12.1 × 10^−4^ and 18.2 × 10^−4^ mg/kg-BW/day for both males and females, respectively. The estimated LCR would be 18 times higher than the upper limit of the U.S. EPA LCR of 1.0 ×10^−4^.

Our cancer risk estimations accounted for “lifetime” exposure to iAs from rice. Although humans may not be consuming rice during infancy, many infant foods are rice-based and contain As. For example, infant rice cereal products sold in the U.S. were shown to have As levels ranging from 0.050 to 0.72 mg/kg, with the lower end of this range being similar to the mean level of iAs found in this study’s rice samples [40]. Importantly, chronic exposure to iAs during early life years may greatly increase one’s lifetime cancer risk [46]. Rasheed et al. took this increased risk into account by using age dependent adjustment factors (ADAFs). The study estimated cancer risk from arsenic in rice to be 1.4 × 10^−3^ among children (ages 6–16) and 8.0 × 10^−4^ among adults (ages 16–67). By using ADAFs, 0–2 year olds acquire 10 times the cancer risk and 2–16 year olds acquire 3 times the cancer risk compared to adults [46]. If we account for an ADAF in our LCR calculation, the average LCR for the 15 and 16 year olds in this cohort will be 15.84 × 10^−4^, which is approximately 16 times higher than the U.S. EPA LCR limit.

### 4.3. Study Strengths and Limitations

As with most of the previously published studies, the limitations of this study include the cross-sectional nature of the study and the lack of long-term exposure history, which restrain our confidence in any causal predictions of arsenic-attributable health risks. Another limitation is the small sample size in both the rice samples collected and number of study participants, which can decrease study power and generalizability to the adolescents living in other parts of China. Additionally, although LCR values across studies could not be confidently compared given that exposure estimation methods are not uniform, the graph in Figure 1 is valuable as it shows overall differences in cancer risks reported by various studies in the published literature.

The bioavailability of arsenic after ingestion of white rice has been measured to be about 40% from animal models and between 55–71% from in vitro studies [8]. Despite this large variation in arsenic bioavailability, it is another factor that should be considered. Furthermore, it is best to base cancer risk calculations on the maximum potential exposure level because it sets the maximum potential risk or “worst case scenario” [1]. We did not assess biomarkers of exposure to As such as in participants’ blood and urine (indicator of short-term exposure) and in hair, skin, or nails (indicator of long-term exposure) [31], which may generate much more accurate exposure and dose-response levels.

As exposure rates from rice can vary day-to-day due to inconsistent diet consumptions, different As concentrations and speciation in rice, rice from different geographical sources, and different rinsing and cooking methods [15]. Cooking rice with iAs contaminated water using methods that result in all cooking water being absorbed into the rice may increase iAs exposure from rice [12]. Although Kunming rice cooking techniques were not analyzed in this study, most Chinese residents cook their rice by simmering with the lid closed until all the water is absorbed. Thorough rinsing and cooking rice in high volumes of water and draining excess water have been found to significantly reduce iAs content in cooked rice [46].

Despite these limitations, this study has distinctive strengths. As displayed in Table 1 and Table 2, many studies use a fraction of iAs from the total arsenic measured to estimate iAs concentrations in rice. The fractions vary considerably, revealing the variability of arsenic speciation in rice and the uncertainty of iAs levels in rice unless directly measured. This study utilized individual-level data which allows for more accurate exposure estimations and directly measured iAs concentrations in rice. Lastly, market rice samples and individual level data are all from a specific region and this is necessary in arsenic exposure estimation due to the many variable factors discussed earlier.

### 4.4. Limitations of Oral Slope Factor

The current U.S. EPA iAs oral slope factor of 1.5 (mg/kg)/day was last revised in 1995 and is based on old studies [50,51] using skin cancer as the only outcome analyzed [5]. The Tseng studies were cross-sectional and analyzed 40,000 Taiwanese exposed to arsenic in drinking water. The researchers found significant excess skin cancer prevalence by comparison to 7500 residents of Taiwan and Matsu who consumed nearly arsenic-free water. Cancer risk estimates were based on water exposure and no data is given on arsenic exposure from food. Additionally, the slope factor was developed using a low-dose extrapolation procedure from higher doses. Reasoning for the use of such old studies are the very large sample size (*N* > 40,000) and the use of a control group that had no evidence of skin lesions or black foot disease for a sensitive endpoint. This is considered superior for developing a reference dose for oral exposure (RfD). Moreover, this data from Taiwan is of value because it removes potential exposure misclassification due to villagers residing in the same location most of their lives, therefore reflecting long-term exposure to regional As levels [52]. Lastly by not taking into consideration internal cancers such as lung and bladder, the oral slope factor for iAs and subsequent cancer calculations may be highly underestimated [5]. Newer oral slope factor proposals are about 17 times greater than the current factor [52].

### 4.5. Public Health Implications

Because China is the highest rice producing and consuming country in the world and accounts for a third of the global supply of rice [9], As-monitoring and enhancing irrigation procedures should be top agricultural priorities in As-affected regions [18]. Future studies should assess changing As levels in agricultural soils over time within the same regions. In particular, the Hunan Province should be a region of focus because Hunan yields roughly 18 million tons of rice annually and includes about 7000 different mines with the potential for environmental contamination [33]. Other important regions include rice growing regions such as Yunnan, Jiangsu, Heibei, Guangdong, Heilongjiang, etc. [18]. There is also a need for increased clinical biomonitoring of high-risk populations in China and more public awareness of the long-term health impacts of iAs and potential exposure from rice. Furthermore, people should be educated about diversifying grains consumed in their diet, washing rice thoroughly before cooking, and cooking rice in higher quantities of water and draining the excess.

## 5. Conclusions

The results of our literature review and analysis reveal that exposure rates and lifetime cancer risk estimations can vary widely between countries, between populations within countries, and between age groups within specific populations. Our study results also suggest that there should be ongoing risk assessments in specific regions, especially in Southern China where residents may be at risk for high exposure levels. Younger populations may be particularly at risk for long-term health effects of chronic iAs exposure from rice because they are highly exposed during earlier years of life and while they are still developing and they have the potential for long-term exposure. In addition, future studies should assess biomarkers of exposure to As such as in participants’ blood, urine, hair, skin, or nails [31]. There are concerns that setting a “too low” MCL could potentially harm the rice industry, but policies need to change if these levels are putting populations at high risk [3]. In perspective, this study cohort’s lifetime cancer risk of 5.28 × 10^−4^ is 5.28 times higher than the U.S. EPA upper limit of 1 in 10,000, with the upper limit being 100 times higher than the universally accepted cancer risk for an environmental carcinogen of one case/one million people [15].

## Figures and Tables

**Table 1:** Summary of studies assessing arsenic concentrations in rice.

Study Location	Author(s) and Year	Source of Rice	Type of Arsenic Measured	Mean (mg/kg) ± SD (Range)
USA	He et al. 2010 [23]	Samples purchased in New York	Total As	0.14 ± 0.0050
Japan	Narukawa et al. 2012 [24]	20 samples from all over Japan	iAs	0.10 (0.056–0.20)
Bangladesh, China, USA	Norton et al. 2012 [25]	6 field trials	Total As	Faridpur: 0.44 (0.19–0.90) Qiyang: 0.68 (0.36–1.27) Arkansas (2006): 0.38 (0.10–0.99) Arkansas (2007): 0.25 (0.030–1.040) Texas (flooded): 0.63 (0.17–1.68) Texas (non-flooded): 0.045 (0.0090–0.13)
China	Sun et al. 2012 [26]	2 samples from market in Guangzhou and rice field in Hunan Province	iAs	0.35 ± 0.0060 (0.40–0.29)
China	Dai et al. 2014 [27]	108 samples from local markets	iAs	Jiangsu: 0.063 Jiangxi: 0.057 Zhejiang: 0.059 Mean of all: 0.059 Range of all: (0.027–0.098)
China	Fang et al. 2014 [18]	92 samples from fields of main rice-growing provinces	Total As	Northern China 0.050 ± 0.040 (* ND–0.13) Southern China 0.11 ± 0.050 (* ND–0.14)
Bangladesh	Ahmed et al. 2016 [21]	10 market samples	Total As	0.32 ± 0.16 (0.14–0.43)
China	Chen et al. 2018 [20]	160 samples from local markets in 20 provinces	iAs	0.054 (0.0090–0.13)

* ND = not detectable.

**Table 2:** Summary of arsenic exposure levels and arsenic-related cancer risk assessments.

Study Location	Author(s) and Year	Age/Group (*N* =)	Source of Arsenic	Type of Arsenic	Mean (mg/kg) ± SD (Range)	As Exposure Estimation (IR = Ingestion Rate of Rice)	EDI (mg/kg-BW/Day)	Cancer Risk
China	Liang et al. 2010 [28]	-	21 rice samples from 13 provinces	iAs	0.082 (0.049–0.22)	IR = 550 g/day (reported from Zhu et al. 2008b) Assuming BW = 60 kg	0.045 mg/day	37.6% contribution to the MTDI (also ^2^ PTDI)
Southern China	Liu et al. 2010 [13]	136 hair & 61 urine samples	33 brown rice samples from Lianhuashan tungsten mining area	Total As (iAs estimated using 83% of total As)	Total As: 0.56 (0.15–1.09)	(IRs from Khan et al. 2008 and Wang et al. 2005) Adult IR = 491.5 g/day Assuming Adult BW = 60 kg Children (1–14 years) IR = 289.6, 232, 92.6 g/day	Adults: 0.23 mg/day	EDI > ^2^ PTDI
India	Mondal et al. 2010 [29]	(*N* = 232) from 3 different villages	Drinking water, cooking water, raw rice, & cooked rice from households	Total As	Raw rice 0.12 ± 0.090 0.16 ± 0.050 0.12 ± 0.020 Drinking water (μg/L) 130 ± 128 40 ± 99 1.0	Water intake Males: 3.1 ± 1.0 L/day Females: 2.6 ± 0.9 L/day Rice IR from National Database of NNMB (2002) Males: 11.60 g/kg/day Females: 11.27 g/kg/day	Water Medians: 2.0 × 10^−5^, 7.7 × 10^−4^, 2.03 × 10^−3^ Cooked rice Medians: 3.0 × 10^−4^, 5.0 × 10^−4^, 8.4 × 10^−4^	(Risk based on total exposure from drinking water, rice, and cooking of rice) LCR Bhawangola-I block: 4.35 × 10^−3^ Chakdha block: 2.04 × 10^−3^ Khejuir-I block: 4.56 × 10^−4^
Ghana	Adomako et al. 2011 [30]	Ages 12 to >40 years (*N* = 204)	549 wholegrain rice samples from Ghana, USA, EU, and Asia	Total As (iAs estimated using 82.9% of total As)	Total As Global mean: 0.14 Ghana: 0.11 ± 0.02 USA: 0.22 ± 0.010 iAs Ghana: 0.091 USA: 0.092 Thailand: 0.10	Ghana IR ≤33.2 to >232.2 g/day Baseline IR = 99.6 g/day, rounded to 100 g/day of dry rice Average BW = 60 kg	Ghana: 9.1 g/day USA: 9.2 g/day Thailand: 10.1 g/day	Used cancer slope factor of 3.67 mg/kg/day (cited by Tsuji et al. 2007): LCR Ghana: 5.57 × 10^−4^ USA: 5.6 × 10^−4^ Thailand: 6.2 × 10^−4^
China	G. Li et al. 2011 [11]	Adults (*N* = 68,962)	494 rice samples	iAs (obtained from regression equation)	North 0.092 ± 0.020 South 0.099 ± 0.042	(Data from China National Nutrition and Health Survey (CNNHS)) North IR = 123.82 g/day South IR = 326.65 g/day (BW = 60 kg used for calculations)	North: 4.7 × 10^−4^ South: 8.8 × 10^−4^	LCR North: 0.76 × 10^−3^ South: 1.31 × 10^−3^
Southern Vietnam	Hanh et al. 2011 [31]	(*N* = 75)	39 rice samples from 45 households	Total As (iAs calculated from total: 80%)	Total As: 0.22 (0.13–0.47)	Males IR = 300 g/day BW = 58 kg Females IR = 250 g/day BW = 50 kg	Males: 0.053 ± 0.018 mg/day Females: 0.045 ± 0.016 mg/day	EDIs were both below the ^1^ BMDL_0.5_
Bengal	Halder et al. 2013 [32]	(*N* = 157)	157 rice samples from households	Total As (fraction of iAs = 0.92)	Total As: 0.010–0.64	18–30 years: 200–400 g/day dry weight 31–50 years: 100–500 g/day 51–65 years: 150–450 g/day	-	When As concentration in drinking water < 10 μg/L, 35% of participants had total daily intake of iAs above ^2^ PTDI
Hunan Province of China	Lei et al. 2013 [33]	Adults & children	34 genotypes of rice grown in As-contaminated field (unpolished rice)	Total and iAs	Total As: 0.42 (0.31–0.52) iAs: 0.39 (0.26–0.52)	Adult IR = 0.40 kg/day (Lin et al. 2004) Children IR = 0.29 kg/day	Adult 0.10–0.21 mg Children 0.080–0.15 mg	Most samples exceeded the ^2^ PTDI
Cambodia	Phan et al. 2013 [34]	Adults	10 rice samples from 3 provinces	iAs assumed to be 80% of total As	Kandal 0.20 ± 0.27 (0.0080–0.95) Kratie 0.064 ± 0.046 (0.0040–0.15) Kampong Cham 0.010 ± 0.0090 (0.0030–0.025)	Rice consumed 3 times/day (approx. 450 g/day) Mean BW = 52 kg	Kandal 1.77 × 10^−3^ (6.80 × 10^−5^–8.23 × 10^−3^) Kratie 5.50 × 10^−4^ (3.70 × 10^−5^–1.32 × 10^−3^) Kampong Cham 8.60 × 10^−5^ (2.80 × 10^−5^–2.10 × 10^−4^)	Kandal: The upper end of the EDI range was greater than the lower limits of ^1^ BMDL_0.5_
Hong Kong	Wong et al. 2013 [35]	20–84 years (*N* = 5008)	600 composite samples of cooked white rice	iAs	0.022 (0.016–0.026)	Food consumption data taken from Hong Kong Population-based Food Consumption Survey (2005–2007)	2.2 × 10^−4^ 95th percentile: 3.8 × 10^−4^	^3^ MOE: 9–32
Zhejiang, China	Z. Huang et al. 2013 [19]	Adults >18 years & children 7–18 years (*N* = 9798)	248 rice samples from local markets in 2012	Total As	0.080 (<LOD **–0.21)	(Food consumption survey from the Zhejiang FDA) Adults IR = 342.90 g/day Adults BW = 55.9 kg Children IR = 258.42 g/day Children BW = 32.7 kg	Adults: 4.9 × 10^−4^ Children: 3.4 × 10^−4^	Health risk index <1.0: no health risk
Southwest Taiwan	Lamm et al. 2014 [36]	Adults >20 years (*N* = 34,783)	Well water from 42 villages	Total As	Means: 10–818 μg/L Range: 10–1752	Village well water data from 1964–1966: Males: 3.5 L/day Females: 2.0 L/day 50 kg BW for both sexes	-	Crude mortality rates (CMR): Maximum value of 2.8 for village with As median of 698 μg/L
Japan	Oguri et al. 2014 [37]	(*N* = 1142)	19 food composites prepared from 159 food items purchased in Shizuoka City	iAs	Raw rice: 0.18 and 0.095	Daily consumption rate of corresponding food category (MHLW 2007): 312.50 g/person/day	Cereals: 0.013 mg/person/day (rice & rice cakes contributed to 97% of iAs intake from cereals)	LCR Skin: 6.1 × 10^−4^ Liver and Lung: 1.2–8.8 × 10^−4^
Europe	Gundert-Remy et al. 2015 [6]	Infants to ≥ 75 years	“All foods”	iAs	0.089 (0.084–0.093)	European Food Safety Authority (EFSA) 2014 survey	Adolescents (10–17 years) Exposure from “all foods”: 1.2 × 10^−4^–4.8 × 10^−4^	Exposure lower than ^1^ BMDL_0.5_
China	Jiang et al. 2015 [38]	2–70 years & over (*N* = 244)	Samples self-cultivated by inhabitants & some from market	Total As (iAs estimated using ratio of iAs/total As 26.8%)	Total: 0.10 (0.046–0.25) iAs: 0.20	Data from Survey of the Nutrition and Health Status (NHS) of the Chinese People in 2002	From all foods: (14–17 years) Males: 1.08 × 10^−4^ Females: 1.01 × 10^−4^	LCR from all foods (14–17 years) Males: 1.62 × 10^−4^ Females: 1.51 × 10^−4^
China	Y. Huang et al. 2015 [9]	2–80 years	1653 rice samples from 11 provinces	iAs	0.091 (* ND–0.30)	IR and BW from Report on Nutrition and Health Status of Chinese Residents (2002)	-	Age 14–18 years: Males: ^3^ MOE=6.28 Females: ^3^ MOE=7.61
Illinois, USA	Bulka et al. 2016 [39]	Males ≥ 15 years (*N* = 4,936,634)	Water	Total As	Mean As tertiles in ppb: 0.33–0.72 0.73–1.60 1.61–16.23	Illinois EPA data on community water systems (2000–2006)	-	Modeling arsenic as a continuous variable: 10 ppb increase in As associated with a 12% increase in SIR for prostate cancer.
USA	Shibata et al. 2016 [40]	Infants 4–24 mo	Rice cereal	iAs	0.091 (0.023–0.28)	(U.S. FDA Rice and Rice Product Sampling (2013) and Signes-Pastor et al. 2016) IR = 14.30–51.50 g/day BW = 6.95–11.85 kg	Median: 9.5 × 10^−6^	LCR Median: 1.4 × 10^−5^
Republic of Kazakhstan, Portugal, and Spain	Tattibayeva et al. 2016 [4]	-	95 rice samples from local markets	iAs	Kazakhstan (unpolished) 0.36 ± 0.020 (0.25–0.45) Spain (milled) 0.25 ± 0.16 (0.15–0.55) Portugal (milled) 0.18 ± 0.15 (0.10–0.30)	Standard adult male BW = 70 kg Children BW = 24 kg	Average Estimated Weekly Intake (EWI) (mg/kg): Kazakh Adults: 7.7 × 10^−4^ Children: 1.88 × 10^−3^ Spain Adults: 2.9 × 10^−4^ Children: 7.0 × 10^−4^ Portugal Adults: 8.4 × 10^−4^ Children: 2.04 × 10^−3^	All EWI values lower than the lower limit of the ^1^ BMDL_0.5_
United States	U.S. FDA 2016 [14]	0–50 years	481 rice samples from retail locations and USA Rice Federation	iAs	White short-grain rice:0.079	Rice intake and each respondent’s BW taken from NHANES/WWEIA (2009–2010)	Mean per capita iAs exposure from rice: 6.6 × 10^−7^	Median estimated total cancer (bladder and lung) cases per million (90% CI) for lifetime: <1 (0, 1.7)
Southern China	Zhuang et al. 2016 [41]	Children & adults	(Long-grain rice) Sample A: market Sample B: mining area Sample C: lab grown	Total As	Sample A: 0.14 ± 0.014 Sample B: 0.17 ± 0.040 Sample C: 0.26 ± 0.077	Adults IR = 389 g/day BW = 60 kg Children IR = 277 g/day BW = 32.5 kg (IR data taken from Wang et al. 2005)	Sample A Adults: 5.3 × 10^−4^ Children: 7.0 × 10^−4^ Sample B Adults: 7.4 × 10^−4^ Children: 1.4 × 10^−3^ (Based on bioaccessible concentrations of As)	Target hazard quotient (THQ): Sample A Adults: 1.77 Children: 2.33 Sample B Adults: 2.48 Children: 4.58 All THQs >1: high non-carcinogenic health risks
Canada	Cheasley et al. 2017 [42]	(*N* = 34,944) from 10 provinces	Rice	Total As (iAs: assumed only 40% of total As)	Total As: 0.065 (0.036–0.094)	Dietary patterns from Health Canada’s Canadian Community Health Survey BW = 70 kg	Urban Median = 1.8 × 10^−3^ mg/day (0.0–0.075) Rural Median = 1.3 × 10^−3^ mg/day (0.0–0.053)	Used cancer slope factor of 1.8: 50–60% of EDIs resulted in LCR values above 10 per million
China	H. B. Li et al. 2017 [8]	Adults	55 rice samples from 15 provinces	Total As	0.13 (0.038–0.34)	Assuming IR of 350 g/day and 60 kg adult	4.1 × 10^−4^–1.5 × 10^−3^	Contributing to 13.7–50.0% of ^1^ BMDL_0.5_
Bangkok	Hensawang et al. 2017 [1]	3–65 years & over	31 rice samples from local markets in 8 different clusters of Bangkok	Total As	0.17 ± 0.0090 (0.084–0.27)	Adolescents (9–19 years): BW = 46.48 kg Consumption per capita = 128.58 g/day	Adolescents: 3.92 × 10^−4^	LCR Adolescents: 2.15 × 10^−8^
Bangladesh	Islam et al. 2017 [43]	Adults	965 rice samples from 73 sub-districts during 2014	iAs	0.20	(Ages 16–19) Males: BW = 52 kg IR = 482 g/day Females: BW = 41.4 kg IR= 453 g/day	-	LCR (Ages 16–19) Males: 2.73 × 10^−3^ Females: 3.22 × 10^−3^
Poland	Mania et al. 2017 [44]	Adults & children	62 rice samples & rice products from trade	iAs	White rice 0.030 90th percentile: 0.060	Data from Central Statistical Office: IR = 0.17 kg/person/month Adult BW = 70 kg Children BW = 20 kg	-	EDI (from rice and rice-based products) ≤ 1% of ^1^ BMDL_0.5_
Ecuador	Nunes et al. 2017 [7]	1–59 years (*N* = 19,932)	16 market basket rice samples & 26 rice samples collected directly from rice paddies	Total As (iAs estimated using ratio of iAs/total As = 0.80 ± 0.08)	Total As Field rice: 0.060 ± 0.052 Market basket rice: 0.070 ± 0.029	IR and BW per age class were based on 24 h recall study of Ministry Health and Nutrition	Men >14 years: 5.4 × 10^−5^ Women >14 years: 6.2 × 10^−5^	LCR Men >14 years: 8.5 × 10^−5^ Women >14 years: 1.0 × 10^−4^
Malaysia	Praveena et al. 2017 [45]	Adults & children	22 varieties of rice from local superstores	Total As	0.091 ± 0.0010	(Values obtained from other studies) Adult IR = 600 g/day BW = 62.65 kg Children (data obtained from Chinese population) IR = 198.4 g/day BW = 19.5 kg	-	LCR Adult: 4.9 × 10^−3^ Children: 3.2 × 10^−3^
Pakistan	Rasheed et al. 2017 [46]	(*N* = 398) 66 children <16 years & 332 adults ≥16 years	Rice	Total As	0.082 ± 0.054	(Data from Rasheed et al. 2016) Ages 6–16 IR = 272 g/day BW = 26 kg Adults >16 years Male IR = 576 g/day Male BW = 68 kg Female IR = 463 g/day Female BW = 55 kg	-	Age 6–16 1.4 × 10^−3^ Age 16–67 8.0 × 10^−4^ Used Age Dependent Adjustment Factors (ADAF)
Northwestern Thailand	Chanpiwat et al. 2018 [47]	Adults	59 locally grown rice samples	iAs estimated assuming 63.2–63.5% of total As is iAs	iAs: 0.20 ± 0.0070 (0.12–0.29)	IR = 84.98 g/day Life expectancy = 75 years Average BW = 63.15 kg	2.0 × 10^−4^ ± 6.6 × 10^−5^ (1.1 × 10^−4^–3.5 × 10^−4^) Bioaccessible As	LCR 4.0 × 10^−−4^ ± 9.0 × 10^−5^ (2.4 × 10^−−4^–6.6 × 10^−4^)

^1^ BMDL_0.5_: iAs lower limit on the benchmark dose for a 0.5% increased incidence of lung cancer = 3.0 × 10^−3^ mg/kg-BW-day [10]; ^2^ PTDI: previous provisional tolerable daily intake recommended by the WHO: 2.1 × 10^−3^ mg/kg-bw/day [21]; ^3^ MOE: ratio of BMDL_0.5_ to iAs dietary exposure. MOE < 100 means significant risk of carcinogenic effects [9]; * ND = not detectable; ** LOD = limit of detection.

**Table 3:** EDI and LCR Calculations.

	Mean BW (kg) ± SD (Range)	ADC (g/Day) ± SD (Range)	AC of iAs (mg/kg) ± SD (Range)	EDI (mg/kg-BW/Day) (Range)	LCR (Range)
Males	67.5 ± 14.4 (44.0–115.9)	409.9 ± 210.7 (0.0–1000.0)	0.058 ± 0.012 (0.045–0.076)	3.52 × 10^−4^ (0–5.00 × 10^−4^)	5.28 × 10^−4^ (0–7.51 × 10^−4^)
Females	55.5 ± 10.6 (37.5–99.4)	336.6 ± 141.0 (50.0–916.7)	0.058 ± 0.012 (0.045–0.076)	3.52 × 10^−4^ (0–5.35 × 10^−4^)	5.28 × 10^−4^ (0–8.02 × 10^−4^)

## Abbreviations

(ADAF)	Age Dependent Adjustment Factor
(As)	Arsenic
(ADC)	Average Daily Consumption Rate of Rice
(BMDL_0.5_)	Benchmark Dose for a 0.5% Increase in Lung Cancer Incidence
(BW)	Body Weight Body Weight Body Weight Body Weight
(EDI)	Estimated Daily Intake of Inorganic Arsenic
(FAO)	Food and Agriculture Organization of the United Nations
(IR)	Ingestion Rate of Rice
(iAs)	Inorganic Arsenic
(AC)	Inorganic Arsenic Concentration
(SF)	Inorganic Arsenic Oral Slope Factor
(IARC)	International Agency for Research on Cancer
(LCR)	Lifetime Cancer Risk
(LOD)	Limit of Detection
(MOE)	Margin of Exposure
(MCL)	Maximum Contaminant Level in China
(ND)	Not Detectable
(PTDI)	Previous Provisional Tolerable Daily Intake
(MOE)	Ratio of BMDL_0.5_ to iAs Dietary Exposure
(U.S. EPA)	United States Environmental Protection Agency
(NHANES)	United States National Health and Nutrition Examination Survey
(WHO)	World Health Organization
